# Engineering Gels with Time-Evolving Viscoelasticity

**DOI:** 10.3390/ma13020438

**Published:** 2020-01-16

**Authors:** Giorgio Mattei, Ludovica Cacopardo, Arti Ahluwalia

**Affiliations:** 1 Department of Information Engineering, University of Pisa, Via Girolamo Caruso 16, 56122 Pisa, Italy; g.mattei86@gmail.com; 2 Research Centre “E. Piaggio”, University of Pisa, Largo Lucio Lazzarino 1, 56122 Pisa, Italy; arti.ahluwalia@unipi.it

**Keywords:** viscoelasticity, dynamic mechanical properties, transglutaminase, ageing

## Abstract

From a mechanical point of view, a native extracellular matrix (ECM) is viscoelastic. It also possesses time-evolving or dynamic behaviour, since pathophysiological processes such as ageing alter their mechanical properties over time. On the other hand, biomaterial research on mechanobiology has focused mainly on the development of substrates with varying stiffness, with a few recent contributions on time- or space-dependent substrate mechanics. This work reports on a new method for engineering dynamic viscoelastic substrates, i.e., substrates in which viscoelastic parameters can change or evolve with time, providing a tool for investigating cell response to the mechanical microenvironment. In particular, a two-step (chemical and enzymatic) crosslinking strategy was implemented to modulate the viscoelastic properties of gelatin hydrogels. First, gels with different glutaraldehyde concentrations were developed to mimic a wide range of soft tissue viscoelastic behaviours. Then their mechanical behaviour was modulated over time using microbial transglutaminase. Typically, enzymatically induced mechanical alterations occurred within the first 24 h of reaction and then the characteristic time constant decreased although the elastic properties were maintained almost constant for up to seven days. Preliminary cell culture tests showed that cells adhered to the gels, and their viability was similar to that of controls. Thus, the strategy proposed in this work is suitable for studying cell response and adaptation to temporal variations of substrate mechanics during culture.

## 1. Introduction

The native extracellular matrix (ECM) is a biphasic material composed of structural elements that confer tissue mechanical strength, stiffness and elasticity, surrounded by an aqueous solution of hyaluronic acid, proteoglycans and glycoproteins, responsible for the viscous behaviour [1,2,3]. Thus, its overall mechanical behaviour is viscoelastic and typically varies over time since biological tissues are continuously subjected to either physiological remodelling processes, such as growth and ageing, or pathological ones—for example, in the case of traumatic events or diseases [4,5,6]. Notably, as the timescales typical of pathophysiological processes are longer than those of the viscoelastic phenomena observed in gels, the evolution of elasticity or viscoelasticity associated with these processes can be studied or mimicked in tissues and materials.

Hydrogels (i.e., highly hydrophilic polymeric networks) have been widely used as ECM mimics for in-vitro cell cultures [1,7]. However, most studies focus only on hydrogel stiffness, which is generally modulated by varying the initial polymer concentration and/or the degree of crosslinking. The materials obtained are assumed to be elastic substrates and to possess constant mechanical properties throughout the culture period. For instance, polyacrylamide (PAAm) gels, the workhorse of mechanobiology studies, are generally characterised by the elastic modulus (*E)* of gels as a function of the relative concentration of acrylamide and bis-acrylamide [8]. Even protein-based hydrogels, which are well known to be viscoelastic, are often represented only in terms of stiffness. Bigi and co-workers showed that the elastic modulus of 5% w/v gelatin hydrogels varied as a function of glutaraldehyde (GTA) [9] and genepin concentrations [10]. In a recent study, Mattei et al. demonstrated that the stiffness of hydroxyapatite/gelatin (Ha/Gel) gels changes as a function of both the HA/Gel ratio and of the GTA [11].

Strategies to alter hydrogel elastic properties over time have been also proposed to study how cells adapt and respond to substrate dynamics. Methods to obtain these dynamic, time-evolving elastic substrates generally use photo-crosslinking approaches based on ultraviolet (UV) light. For example, the *E* of polyethylene (PEG)-thiol gels was increased from 0.24 up to 12 kPa thanks to in-situ photo-polymerization using lithium phenyl-trimethyl-benzoyl phosphonate (LAP) as a photo-initiator [12]. Similarly, thiolated hyaluronic acid (HA)/polyethylene glycol diacrylate (PEGDA) hydrogels showed a significant increase in stiffness as a function of the post-polymerization time [13]. However, besides the possible phototoxicity of UV light, the addition of photopolymerization initiators can produce cytotoxic free-radicals. For this reason, Stowers and co-workers proposed a different approach, based on the use of near-infrared (NIR) light in association with temperature-sensitive liposomes that triggered the release of calcium to stiffen alginate gels [14]. The kinetics of chemical or enzymatic reactions can also be used to modulate hydrogel crosslinking and thus their stiffening over time, again resulting in dynamic or time-evolving elastic substrates. In particular, reaction kinetics depend on the polymer structure, composition and molecular weight, as well as on the ratio between substrate and enzyme concentration and other environmental conditions, such as temperature and pH [15,16,17]. For example, using Michael-type addition, the stiffness of thiolated-HA hydrogels crosslinked with PEGDA can be modulated over the typical time scales of physiological process, such as embryonic development [18].

While the role of stiffness in orchestrating cell behaviour has been widely studied, the effect of viscoelastic properties, or cell response to viscotaxis [19] is still scarcely investigated [20,21]. Some recent reports describe how gel viscoelastic properties have been modulated both by varying the polymer concentration, molecular weight and crosslinking [22,23,24] or by altering the liquid phase viscosity [25] to obtain viscoelastic substrates (i.e., with a time dependent behaviour characterised by constant viscoelastic properties over the culture period). Charrier and colleagues showed that the loss modulus (G″) of PAAm gels can be modulated by entrapping high molecular weight linear polymers in the gels [23]. Similarly, varying the proportion of acrylamide monomer and bis-acrylamide resulted in different loss moduli [22]. Moreover, Chaudhuri and co-workers demonstrated that the characteristic relaxation time of alginate gels can be modified by varying the polymer molecular weight or with the addition of PEG spacers to provide a steric hindrance to alginate crosslinking [24]. Finally, the relaxation time of agarose and PAAm gels was modulated by varying the viscosity of the gel liquid phase with different dextran concentrations while maintaining a constant equilibrium modulus [25]. 

In the light of these considerations, it is clear that dynamic viscoelastic substrates (i.e., with viscoelastic properties that evolve over time) are needed to fully recapitulate native ECM mechanical properties in vitro. To date, only a few examples of such materials can be found in the literature. For instance, magneto-active hydrogels were prepared by Abdeen and colleagues, incorporating carbonyl iron particles into polyacrylamide gels. In particular, both the shear elastic and storage moduli (G′ and G″) increased in response to an external magnetic field ramp [26]. Moreover, Rodell et al. demonstrated that Michael-type additions in thiolated HA gels can be associated with an alteration of both G′ and G″ and that the reaction kinetics can be modulated by controlling Michael-acceptor reactivity and catalytic conditions [27]. Furthermore, as reported by Guvendiren and Burdick, photopolymerization of methacrylate hyaluronic acid (Me-HA) resulted in an increase of both G′ and G″ suggesting a soft-to-stiff transition [28]. Conversely, a stiff-to-soft transition was obtained by photodegradation of HA gels modified with o-nitrobenzyl-acrylate and methacrylate [29]. 

To summarise, cell culture substrates can manifest different mechanical behaviour which can be quantified through one or more constitutive parameters to describe their properties [29]. On the basis of the state of the art described above, we can classify substrates as

1. Elastic—these substrates are described by a constant elastic modulus, *E*, and represents the classical tool for mechanobiology studies in-vitro;
2. Viscoelastic, time-dependent behaviour described by one or more elastic moduli, *E*, and viscous moduli, *η_i_*, with values that are constant over time;
3. Dynamic elastic, described by an elastic modulus, *E(t)*, which change or evolves as a function of time;
4. Dynamic viscoelastic, i.e., with viscoelastic properties which evolve over time and are thus described by one or more elastic moduli *E_i_(t)*, and viscosities, *η_i_(t).*

Table 1 recaps these concepts.

In this study, we describe a novel method for engineering cell culture substrates able to mimic dynamic, time-evolving viscoelastic transitions during culture. In particular, a two-step crosslinking procedure was employed to modulate the mechanical behaviour of gelatin hydrogels, which are typically used as a cell culture substrates [30]. The hydrogels were firstly crosslinked using glutaraldehyde (GTA), obtaining stable gels at physiological temperature (i.e., 37 °C). Then their viscoelastic properties were modulated through a cytocompatible crosslinking mediated by microbial transglutaminase (mTG). This enzyme catalyses the formation of covalent bonds between glutamine and lysine in several vegetable and animal organisms including humans [17,31,32], and has been widely used to improve the mechanical strength of gelatin and collagen-based scaffolds in the presence of cells [31,32,33,34,35,36,37]. The time-evolving viscoelastic properties of these substrates were characterised over 7 days at typical cell length-scales using nanoindentation tests [38]. 

## 2. Materials and Methods 

### 2.1. Sample Preparation and First Step (Chemical) Crosslinking

A 5% w/v gelatin solution was prepared by dissolving type A gelatin powder (G2500, Sigma-Aldrich, Milan, Italy) in 1Xphosphate buffered saline (PBS 1X, Sigma-Aldrich) at 50 °C under stirring for 2 h. Cylindrical gelatin samples with ~ 3 mm thickness were obtained by casting 3 mL of the solution into 35 mm diameter Petri dishes (Corning Inc. - Corning, NY, USA) and left to physically crosslink for 2 h at 4 °C. Then, they were exposed to UV light for 20 min (while on ice). Subsequently, 3 mL of glutaraldehyde (GTA; G5882, Sigma-Aldrich) solutions prepared at different concentrations (i.e., 1.25, 2.5, 5, 10, 25, 50 mM) in 40% v/v ethanol/water were added to the samples which were maintained for 48 h at 4 °C for chemical crosslinking. After washing thrice with PBS, samples were incubated in 3 mL of 200 mM glycine solution (G7126, Sigma-Aldrich) in a humidified cell culture incubator (37 °C, 5% CO_2_) for 1 h to quench unreacted GTA moieties. The samples were then subjected to further rinsing with PBS and ethanol and overnight incubation in cell culture medium (Dulbecco’s Modified Eagles Medium or DMEM, Sigma-Aldrich). Finally, the medium was discarded, and the samples were washed thrice with PBS and readied for mechanical testing (day 0).

### 2.2. Second Step (Enzymatic) Crosslinking

GTA-crosslinked samples were exposed to the second step enzymatic amino-crosslinker in order to alter their viscoelastic properties over time. Briefly, after overnight preconditioning in the incubator (day 0), the samples were washed and submerged in 3 mL of microbial-transglutaminase (mTG ACTIVA^®^ WM kindly supplied by Ajinomoto Foods Europe SAS, Mesnil-Saint-Nicaise, France) solutions prepared at different concentrations (namely 1, 10 and 100 U/g, i.e., enzyme units per dry gram of gelatin polymer to crosslink) in PBS 1X. Samples were then incubated at 37 °C/5% CO_2_ for up to seven days and characterised through nanoindentation (Section 2.4) after 1, 4, and 7 days. Samples submerged in PBS without mTG were used as a control. 

### 2.3. Viscoelastic Parameter Estimation

**Figure 1:**
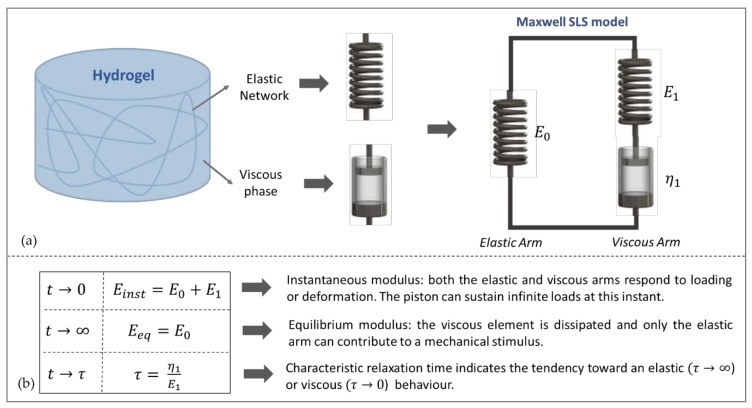
Representing hydrogel viscoelasticity with lumped parameter models: (**a**) The Maxwell-type Standard Linear Solid (SLS) model; (**b**) relationships between the lumped parameter elements and the instantaneous (*E_inst_*) and equilibrium (*E_eq_*) elastic moduli and the characteristic relaxation time (τ).

In a biphasic material such as a hydrogel, the elastic network can be represented as a spring and the viscous phase as a dashpot. A combination of these elements can be used to model hydrogel viscoelasticity. In this work, a Maxwell-type Standard Linear Solid (SLS) model was chosen since it has been shown to well represent the viscoelastic behaviour of gelatin based hydrogels [38,39,40,41]. The SLS model is the simplest form of the Generalized Maxwell (GM) lumped parameter model. It consists of a pure spring ($E_{0}$) in parallel with a Maxwell arm (i.e., a spring $E_{1}$ in series with a dashpot $\eta_{1}$) [42] and is shown in Figure 1a. 

As outlined in Figure 1b, $E_{inst}$ and $E_{eq}$ represent, respectively, the initial response of the system for *t* *→* 0 and the equilibrium response for *t* *→ ∞.* Initially, the dashpot can sustain an infinite load and can be considered to be ‘shorte’,” and both arms contribute to the mechanical response. At equilibrium, the dashpot is completely dissipated and the viscous arm can be thought of as ‘open’. Thus, only the elastic arm represented by E_0_ contributes to the mechanical behaviour of the system. The time constants can be used to compare the viscoelastic behaviour of materials. A high τ indicates that the material presents a tendency towards an elastic or ‘solid-like’ behaviour; conversely, a low τ indicates a tendency toward viscous or ‘liquid-like behaviour’. In particular, when τ *→ ∞* the material is referred as pure elastic and when τ *→* 0 it is considered as pure viscous [25,43].

### 2.4. Nano-Indentation Measurements

Sample viscoelastic properties were characterised using the nano-epsilon dot method (nano-$\dot{\varepsilon }M$), which is based on performing nano-indentation measurements at different constant strain rates [38]. In particular, the samples were submerged in fresh PBS 1X at 37 °C and tested directly in their Petri dishes. Nano-indentation measurements were performed at 37 °C at five different constant indentation strain rates (${\dot{\varepsilon }}_{ind}$ = 0.01, 0.025, 0.05, 0.1, 0.25 s^−1^) using a PIUMA Nanoindenter (Optics11 B.V., Amsterdam, The Netherlands) equipped with a 49.5 µm radius (*R*) spherical probe and 0.460 N/m cantilever stiffness (*k*) and a temperature controlled sample stage. The experimental indentation velocity ($\dot{h}$) to obtain the desired constant strain rate was calculated as follows (Equation (1)) [38,44]:

(1) $$\dot{h}=\frac{3}{4}\times \left(1-\upsilon^{2}\right)\times R\times {\dot{\varepsilon }}_{ind},$$

where υ denotes the Poisson’s ratio, here assumed equal to 0.5 (i.e., incompressible material) for gelatin-based hydrogels [30,38,45,46]. 

Three hydrogel replicates (n = 3) were prepared per combination of GTA (first-step crosslinker) and mTG (second-step crosslinker) concentration used. Samples were treated as mechanically isotropic [30,38,44] and tested at different time points (i.e., day 0, 1, 4, and 7) by performing measurements on n = 9 (randomly selected) surface points per strain rate investigated. Nano-indentation measurements were started out of sample contact and performed at different locations on the surface to avoid pre-stress and effects due to repeated testing cycles on the same point. 

### 2.5. Nanoindentation Data Analysis

Only data belonging to the loading portion of the load-indentation (*P-h*) curves measured at different $\dot{\varepsilon }$ were analysed. The initial contact point was identified as the last one at which the load crosses the *P-h* abscissa towards monotonically increasing values [38,41]. Experimental *P-h* time data were offset to be zero in correspondence with this point. Load-indentation data were converted respectively into indentation stress ($\sigma_{ind}$) and strain ($\varepsilon_{ind}$) according to Equations (2) and (3) [38].

(2) $$\sigma_{ind}=\frac{P}{R\times \sqrt{hR}}$$

(3) $$\varepsilon_{ind}=\frac{4}{3\times \left(1-\upsilon^{2}\right)}\times \frac{h}{R}$$

For each sample and experimental time point, stress-strain data obtained from the three hydrogel replicates tested at the same ${\dot{\varepsilon }}_{ind}$ (n = 27 dataset) were pooled together, computing their mean and standard error of the mean (SEM). The sample linear viscoelastic region (LVR) was identified as the strain region in which $\sigma_{ind}$ increases linearly with $\varepsilon_{ind}$, returning a R^2^ ˃ 0.99 [30]. 

The Maxwell SLS model depicted in Figure 1a exhibits the following stress-time response to a constant indentation strain rate input ${\dot{\varepsilon }}_{ind}$ (Equation (4)) [38,41]:

(4) $$\sigma_{ind}\left(t\right)={\dot{\varepsilon }}_{ind}\times \left(E_{0}t+\eta_{1}\times \left(1-e^{-\frac{E_{1}}{\eta_{1}}t}\right)\right).$$

For each sample and experimental time point, average stress-time data belonging to the sample LVR (along with their SEM) obtained at different ${\dot{\varepsilon }}_{ind}$ were globally fitted to Equation (4) for deriving the Maxwell SLS viscoelastic constants (i.e., $E_{0}$, $E_{1}$ and $\eta_{1}$). The global fitting procedure was implemented in OriginPro (OriginLab Corp., Northampton, MA, USA), performing chi-square minimization in a combined parameter space. In particular, for each stress-time dataset considered in the global fitting, the ${\dot{\varepsilon }}_{ind}$ value in Equation (4) was set equal to that used in experiments, while the SLS viscoelastic constants to estimate were shared between datasets. 

We also used a more complex model with two viscous arms, but there was no improvement in the error between the data and fitted equation. Moreover, the time constants were equivalent for both arms suggesting over parametrization. Previous studies corroborate this finding [43].

An annealing scheme based on multiplying and dividing each initial parameter guess by 10 while keeping the instantaneous modulus (i.e., $E_{inst}=E_{0}+E_{1}$) at a constant value was adopted to obtain reliable and absolute SLS viscoelastic constant estimations, avoiding local minima during the fitting procedure. Viscoelastic constants to estimate were constrained to be ≥ 0 to prevent the fitting procedure returning negative values. The results obtained were used to calculate the instantaneous and equilibrium $E_{eq}=E_{0}$ elastic moduli as well as the characteristic relaxation time ($\tau =\eta_{1}/E_{1}$) for each sample investigated at each different time point. 

### 2.6. Gel Degradation Tests

Protein release in 5 mM GTA hydrogels crosslinked with 100 U/g mTG was assessed to determine whether GTA and mTG-GTA hydrogels differ in terms of their degradation rates. Briefly, the gels were prepared as reported above (Section 2.1 and Section 2.2) and samples were collected from the supernatant at day 1 and 7. The Bicinchoninic acid (BCA) assay (23227, ThermoFisher, Waltham, MA, USA) was used to quantify the relative protein content released from the gels. Absorbance was read at a wavelength of 592 nm with an OMEGA FLUOstar Spectrophotometer (BMG LabTech, Ortenberg, Germany). Uncrosslinked gelatin and GTA crosslinked hydrogels without mTG were used as controls. Gel degradation was related to the percentage of protein mass released with respect to the initial mass of gelatin in the samples.

### 2.7. Cytocompatibility Tests

At day 0, adipose derived mesenchymal stem cells (aMSC, a kind gift from Professor Anna Maria Bassi, University of Genova, Genova, Italy) were seeded on the 5 mM GTA-gels at a density of 50,000 cells/cm^2^. The cell culture medium was DMEM supplemented with 10% FBS, 1% L-Glutamine, 1% non-essential amino acids (NEAA), and 1% Penicillin/Streptomycin. At day 1, the medium was supplemented with 100 U/g of mTG sterilised by filtration, and the cells were cultured for up to seven days. Cells cultured on polystyrene wells in the same conditions (i.e., with 100 U/g of mTG dissolved in the medium after the first day of culture) were used as controls.

Cell viability was assessed on n = 3 different gels and on n = 3 well controls with the Alamar blue assay at day 0 and day 7. Briefly, a 10% resazurin solution was prepared in complete culture media and incubated with the hydrogels for 6 h at 37 °C. Then, 100 µL was collected in triplicate for each sample and analysed in a fluorescence spectrophotometer (OMEGA FLUOstar, BMG LabTech GmbH – Ortenberg, Germany) using an excitation wavelength of 490 nm and an emission wavelength of 610 nm. Results were expressed in terms of relative viability normalising data with respect to day 0. Finally, cells were fixed with 4% w/v paraformaldehyde (PFA) and permeabilized with 0.1% Triton. Cell nuclei were stained with DAPI and actin with red alexa fluor 594-conjugated phalloidin (ThermoFisher, Waltham, MA, USA). Images were acquired with a confocal microscope (Nikon A1, Tokyo, Japan). Unless otherwise specified, all cell culture reagents were purchased from Sigma-Aldrich (Milan, Italy).

### 2.8. Statistical Analysis

Results are reported as mean ±SEM. Statistical differences between viscoelastic parameters obtained for gelatin hydrogels were tested using one-way (day 0; variability factor: GTA concentration) or two-way (day 1, 4, 7; variability factors: mTG concentration and time) Analysis of Variance (ANOVA) followed by Tukey’s Multiple Comparison Test.

Differences in viability (gel vs. well) and gel degradation (day 1 vs. day 7) were tested using the Student’s t-test.

All statistical analyses were performed using GraphPad Prism (GraphPad Software, San Diego, CA), setting significance at *p* < 0.05. To improve readability, estimated viscoelastic parameters are presented as graphs in the paper while the data and ANOVA results are available in the Supplementary Materials. 

## 3. Results

### 3.1. Initial Hydrogel Viscoelastic Properties (Day 0)

**Figure 2:**
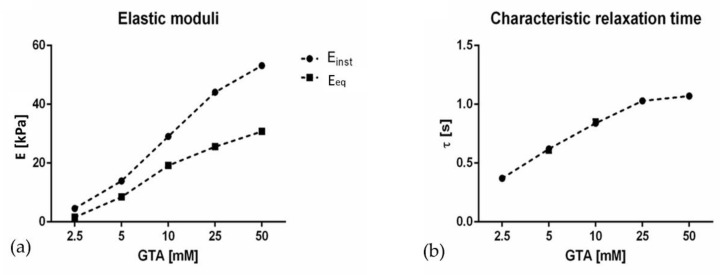
(**a**) Instantaneous *E_inst_*) and equilibrium (*E_eq_*) elastic moduli and (**b**) characteristic relaxation times of gelatin hydrogels as a function of glutaraldehyde (GTA) concentration. The dashed lines represent linear data interpolation and serve only as a guide to the eye. Error bars are scarcely visible due to their very low values (SEM values are reported in the Supplementary Materials).

Figure 2 shows the viscoelastic parameters obtained for gelatin hydrogels after the first step of chemical crosslinking with GTA (i.e., day 0). The graphs show that increasing GTA concentration not only results in hydrogel stiffening, as indicated by the increased $E_{inst}$ and $E_{eq}$ and as expected from the literature [9,11], but there is also a concomitant change in their viscoelastic behaviour toward a more elastic one, as indicated by the longer relaxation time [30]. One-way ANOVA analysis showed that the increase in both elastic moduli and characteristic relaxation time were all significant with increasing GTA concentration (*p* < 0.0001), with the exception of the characteristic relaxation time between 25 and 50 mM GTA-crosslinked hydrogels (*p* = 0.74, see Supplementary Materials).

Notably, gelatin hydrogels crosslinked with 1.25 mM GTA were not stable at 37 °C due to their low degree of crosslinking. The monotonic sample stiffening observed with increasing GTA concentration reflects an increase in the degree of crosslinking between gelatin-free amino groups and GTA aldehydes. Moreover, the absence of $E_{inst}$ and $E_{eq}$ plateaus suggests that gelatin amino groups were not saturated at the GTA concentrations used, leaving unreacted substrate for the second-step enzymatic amino-crosslinking.

### 3.2. Evolution of Hydrogel Viscoelastic Properties

**Figure 3:**
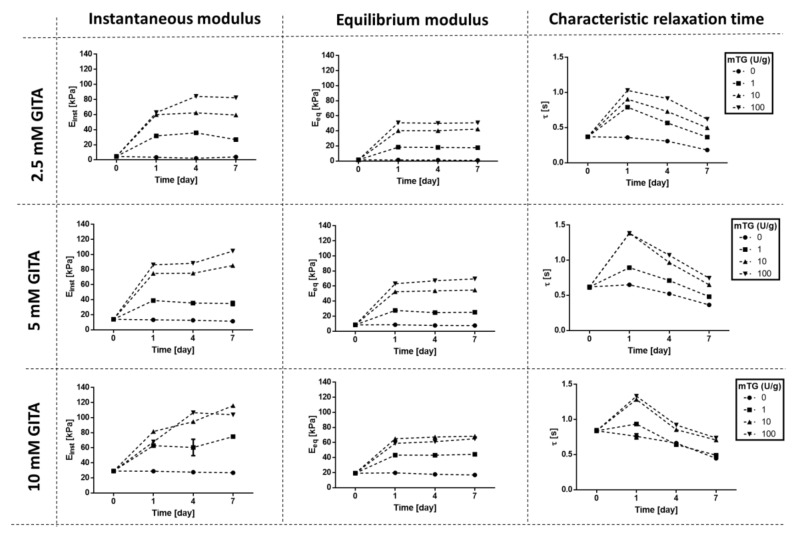
Instantaneous (*E_inst_*) and equilibrium (*E_eq_*) elastic moduli and characteristic relaxation times of GTA-crosslinked gelatin hydrogels incubated with mTG for 1, 4, and 7 days. Error bars are scarcely visible due to their very low values (SEM values are reported in the Supplementary Materials).

Temporal viscoelastic changes in response to the second-step (enzymatic) crosslinker were characterised only for gelatin hydrogels crosslinked with 2.5, 5 and 10 mM GTA, since their initial viscoelastic properties (shown in Section 3.1) lie in the typical physiological range observed for several soft tissues [47,48,49,50]. Viscoelastic parameters obtained by incubating these samples for 1, 4, and 7 days in 1, 10, and 100 U/g mTG enzymatic solutions are summarized in Figure 3, along with the parameters obtained for the controls in PBS.

Control samples (incubated in PBS only, 0 U/g mTG) were fairly stable over time, as indicated by the almost constant $E_{inst}$ and $E_{eq}$ values, especially at 5 and 10 mM GTA concentrations. A slight drop in $E_{eq}$ was observed for 2.5 mM samples, as expected, because of their low degree of crosslinking. Moreover, we observed a gradual decrease of the characteristic relaxation time τ for the control gels, indicating that the gels gradually become more viscoelastic and less elastic. 

All GTA-crosslinked samples were significantly stiffened by the second-step enzymatic crosslinking with mTG, as indicated by the increase in $E_{inst}$ and $E_{eq}$ after day 0. This stiffening was accompanied by a shift towards a more elastic behaviour (indicated by the longer relaxation time), similar to that observed with increasing GTA concentration in the first-step chemical crosslinking. As in the single step GTA crosslinked gels, the almost constant $E_{eq}$ values from day 1 onwards in the gels exposed to GTA and mTG indicate that an intrinsic mechanical stability was maintained during the experiments. 

Sample viscoelastic properties were principally altered during the first day of enzymatic incubation. Subsequently, $E_{inst}$ and $E_{eq}$ were generally maintained almost constant over time, with the exception of gels in the presence of the highest concentration of mTG (100 U/g), where a significant increase of $E_{inst}$ was observed over time, independent of the initial GTA crosslinker concentration. A general decrease in the characteristic relaxation time was observed from day 1 to day 7 for all samples, regardless of the GTA and mTG concentrations, indicating a shift back to a more viscous behaviour. This shift was more marked at higher mTG concentrations.

### 3.3. Gel Degradation

Gel degradation, expressed as the percentage of protein content in the supernatant with respect to the initial protein content, is reported in Table 2. Both at day 1 and 7, the degradation was significantly lower in the mTG-GTA crosslinked gels with respect to the GTA crosslinked controls. For both gels, the data at day 7 were significantly higher than at day 1. However, the relative increase was higher for the mTG-GTA gels. As expected, un-crosslinked gelatin dissolved after 1 day at 37 °C [36,37]. 

### 3.4. Cytocompatibility 

**Figure 4:**
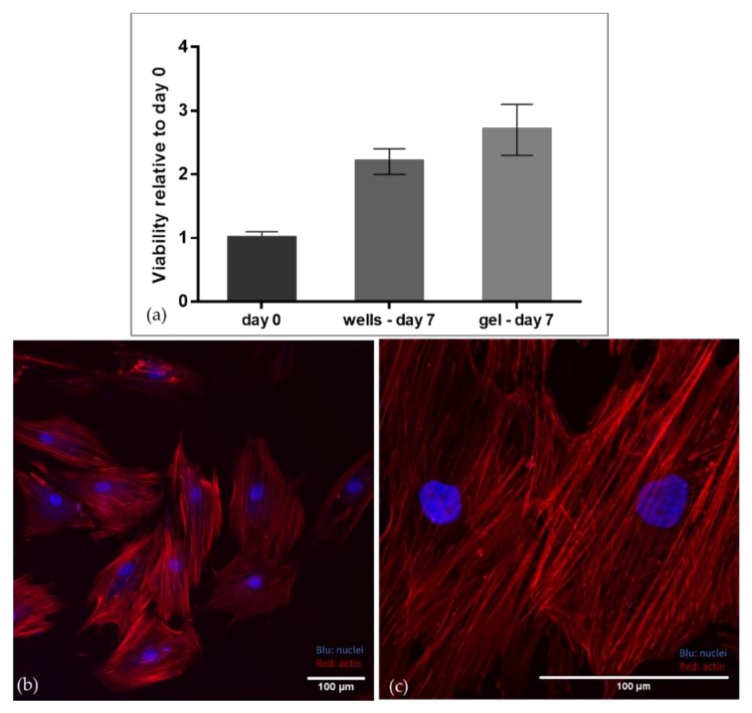
(**a**) Cell viability relative to day 0; (**b**) 20X and (**c**) 20X Nyquist confocal images of aMSC on the mTG-GTA gels after seven days of culture. Nuclei are stained with DAPI (blue) and actin is labelled with Alexa 594-conjugated phalloidin (red).

As shown in Figure 4a, no significant differences in cell viability were observed on the gels with respect to polystyrene wells. Figure 4b,c demonstrate that cells adhere and thus spread on the gels expressing well-defined actin fibers.

## 4. Discussion

As outlined in Table 1, the mechanical behaviour of cell culture substrates used to study mechanobiology can be classified into four groups according to whether their elasticity or viscoelasticity evolves with time or not. In this paper, a two-step crosslinking strategy using GTA and mTG-crosslinked gelatin was used to engineer dynamic, time-evolving viscoelastic cell culture substrates with viscoelastic properties which change over time.

**Figure 5:**
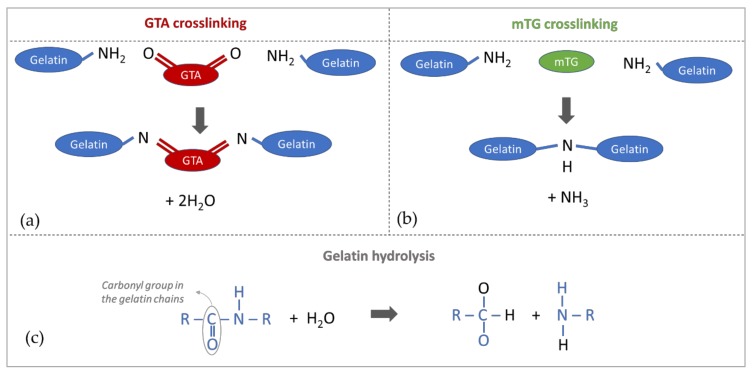
Schematic of gelatin crosslinking with (**a**) glutaraldehyde (GTA) and (**b**) microbial transglutaminase (mTG); (**c**) Schematic of gelatin hydrolysis reaction.

The differences between the first crosslinking step with GTA and the second one with mTG are highlighted in Figure 5a,b. In both cases, gelatin chains are crosslinked thanks to the formation of covalent bonds between gelatin amino residues (NH_2_). In particular, GTA molecules interpose themselves between amino residues in gelatin chains, while mTG catalyses the direct binding between two amino groups [51,52]. 

The first chemical crosslinking step with GTA provided stable hydrogels with equilibrium elastic moduli ranging from few kPa up to ~30 kPa. However, only gels with 2.5, 5 and 10 mM GTA matched the mechanical properties typical of ‘healthy’ soft tissues (within ~20 kPa [4]). The second step using mTG allowed the modulation of gelatin viscoelastic properties in conditions compatible with cell cultures, i.e., at 37 °C and without the use of cytotoxic reagents. Enzyme-mediated crosslinking allowed us to mimic a pathophysiologic mechanical transition, increasing the gel equilibrium modulus by up to ~80 kPa. The mechanical stiffening occurred within 24 h and subsequently the equilibrium modulus (generally associated with ‘stiffness’) was maintained almost constant until day 7, a typical timeframe for observing cell cultures. In particular, in the first step, the increase of glutaraldehyde concentration induced an increase of both the instantaneous and equilibrium modulus and of the characteristic relaxation time, indicating that the gels become stiffer and more elastic (hence less viscous). Similarly, 24 h after the application of mTG (at day 1), we observed an increase of the elastic moduli and of the relaxation time. This trend is expected because both reactions are associated with a higher number of covalent crosslinks between gelatin amino-groups [53]. Between 1 and 7 days after the application of mTG, the equilibrium modulus remained constant, while the relaxation time decreased. This suggests that the substrate maintains a stable structure but with a shift towards a more viscous or ‘liquid-like’ behaviour. We hypothesise that the covalent bonds in the chemically crosslinked network are the main contribution to the equilibrium modulus and the increase in viscous behaviour one day after exposure to mTG is probably due to gel degradation related to hydrolytic phenomena. Hydrolysis has been reported to affect the carbonyl groups in the polymer chains (Figure 5c) rather than the amino groups involved in the GTA or mTG mediated crosslinking (Figure 5a,b) [54,55]. Consequently, the more mobile hydrolysed gelatin residues may affect the relaxation time without significantly changing $E_{eq}$.

Gel degradation tests on GTA and mTG-GTA crosslinked gels support this hypothesis. The higher relaxation time of the hydrogels crosslinked with mTG and GTA is reflected in their lower degree of degradation with respect to GTA-crosslinked hydrogels. In particular, the τ in latter gels decreases at a slower rate than the two step GTA-mTG gels, which is reflected in the difference between the degradation of the gels at day 1 and 7. Similarly, the rapid decrease in τ of the mTG-GTA gels from day 1 to 7 can be associated with the higher difference between degradation at day 1 and 7 for these samples. 

The observed time-evolving viscoelastic behaviour has been also found in different tissues. For example, in tendons, beside the increase of elastic modulus, a significant decrease in viscosity was observed both for the fibers and for the embedding matrix as a function of ageing [56]. Similarly, skin becomes less elastic and more viscous with age, as highlighted by the decrease of the relaxation time and of recovery capacity [57,58]. Finally, it was found that brain viscosity is subject to a continuous decline over time, resulting in an increase of the ‘liquid-like’ behaviour [59]. Indeed, in ageing and diseases, concurrently with the stiffening due to glycation or lysyloxidase (LOX) upregulation [4,60], the depletion and degradation of components, such as glycosaminoglycans and hyaluronic acid, is likely to reduce the viscosity (and hence decrease the resistance to flow) of interstitial fluids [57,61,62]. 

The cytocompatibility of gelatin crosslinked with mTG and GTA has already been demonstrated in the literature [37]. We performed preliminary tests to assess the cytocompatibility of gelatin subjected to both crosslinking reagents. In particular, mTG was administered as a second step crosslinking reagent after cell seeding. Our results confirmed that the combination of the two crosslinking agents is cytocompatible. Indeed, the gels demonstrated good adhesive properties and cell viability was comparable with cells cultured on standard tissue culture plates. 

In conclusion, the proposed strategy enables the study of cell adaptation to a mechanically changing environment. Cells can be exposed to the enzyme ‘on-demand’ at any time during culture, in order to study their response to a sudden increase in substrate stiffness (i.e., increase $E_{eq}$) and elasticity, and to a progressive increase of the viscous or liquid-like behavior typical of pathophysiological processes associated with ageing.

## Figures and Tables

**Table 1:** Summary of cell culture substrate mechanical properties and their time dependence (or viscoelasticity) and time evolution (or change in properties with time).

	Change in Properties with Time	Viscoelasticity	Parameter(s):	Examples of Typical Substrates	Refs
**Elastic**	no	no	*E*	GTA or genepin crosslinked gelatin, Ha/Gel, PAAm	[8,9,10,11]
**Viscoelastic**	no	yes	*E_i_*, *η_i_*	PAAm, alginate, agarose	[22,23,24,25]
**Dynamic/time-evolving Elastic**	yes	no	*E (t)*	PEG-thiol, HA/PEGDA, Ca^2+^-liposome loaded alginate	[12,13,14,18]
**Dynamic/time-evolving Viscoelastic**	yes	yes	*E_i_ (t)*, *η_i_ (t)*	Magneto active PAAm, HA-based gels	[26,27,28,29]

**Table 2:** Gel degradation expressed as the percentage of protein content in the supernatant with respect to the initial protein content.

	Day 1	Day 7
*GTA crosslinked gels (controls)*	8.3% ± 0.7% ^a^	10.3% ± 0.6% ^c^
*mTG-GTA crosslinked gels*	2.1% ± 0.3% ^b^	6.2% ± 0.2% ^d^

^a, b, c, d^: Different letters indicate significant differences.

## Supplementary Materials

Data plotted in the graphs in the main text and the results of the statistical are available online at https://www.mdpi.com/1996-1944/13/2/438/s1.
