# Supplementary material for: Engineering Gels with Time-Evolving Viscoelasticity
Source: Materials (Basel). 2020 Jan 16;13(2):438. doi: 10.3390/ma13020438 (PMC7014018; doi:10.3390/ma13020438)
Supplement: Supplementary file 1 [file materials-13-00438-s001.pdf]

materials

Supplementary Materials

Engineering Gels with Time-Evolving Viscoelasticity

The following tables report the data plotted in the graphs in the main text and the results of the statistical analyses (significant differences are highlighted in green).

1. First (chemical) crosslinking step (day 0)

	2.5 mM		5 mM		10 mM		25 mM		50 mM	
	Mean	SEM	Mean	SEM	Mean	SEM	Mean	SEM	Mean	SEM
E_{inst} (Pa)	4604	60	13920	111	29090	166	44110	221	53180	135
E_{eq} (Pa)	1564	4	8480	68	19165	43	25573	66	30745	66
τ (s)	0.37	0.01	0.62	0.03	0.84	0.03	1.03	0.02	1.07	0.02

Instantaneous modulus (E_{inst})

Tukey's multiple comparison test					
	Mean Diff	95% CI of diff	Significant?	Summary	Adjusted P Value
2.5 vs. 5 mM	−9316	−9898 to −8734	Yes	****	< 0.0001
2.5 vs. 10 mM	−24486	−25068 to −23904	Yes	****	< 0.0001
2.5 vs. 25 mM	−39506	−40088 to −38924	Yes	****	< 0.0001
2.5 vs. 50 mM	−48576	−49158 to −47994	Yes	****	< 0.0001
5 vs. 10 mM	−15170	−15752 to −14588	Yes	****	< 0.0001
5 vs. 25 mM	−30190	−30772 to −29608	Yes	****	< 0.0001
5 vs. 50 mM	−39260	−39842 to −38678	Yes	****	< 0.0001
10 vs. 25 mM	−15020	−15602 to −14438	Yes	****	< 0.0001
10 vs. 50 mM	−24090	−24672 to −23508	Yes	****	< 0.0001
25 vs. 50 mM	−9070	−9652 to −8488	Yes	****	< 0.0001

Equilibrium modulus (E_{eq})

Tukey's multiple comparison test					
	Mean Diff	95% CI of diff	Significant?	Summary	Adjusted P Value
2.5 vs. 5 mM	−6916	−7132 to −6700	Yes	****	< 0.0001
2.5 vs. 10 mM	−17601	−17817 to −17385	Yes	****	< 0.0001
2.5 vs. 25 mM	−24009	−24225 to −23793	Yes	****	< 0.0001
2.5 vs. 50 mM	−29181	−29397 to −28965	Yes	****	< 0.0001
5 vs. 10 mM	−10685	−10901 to −10469	Yes	****	< 0.0001
5 vs. 25 mM	−17093	−17309 to −16877	Yes	****	< 0.0001
5 vs. 50 mM	−22265	−22481 to −22049	Yes	****	< 0.0001
10 vs. 25 mM	−6408	−6624 to −6192	Yes	****	< 0.0001
10 vs. 50 mM	−11580	−11796 to −11364	Yes	****	< 0.0001

25 vs. 50 mM	−5172	−5388 to −4956	Yes	****	< 0.0001

Relaxation time τ (s)

Tukey's multiple comparison test					
	**Mean Diff**	**95% CI of diff**	**Significant?**	**Summary**	**Adjusted P Value**
2.5 vs. 5 mM	−0.25	−0.3409 to −0.1591	Yes	****	< 0.0001
2.5 vs. 10 mM	−0.47	−0.5609 to −0.3791	Yes	****	< 0.0001
2.5 vs. 25 mM	−0.66	−0.7509 to −0.5691	Yes	****	< 0.0001
2.5 vs. 50 mM	−0.7	−0.7909 to −0.6091	Yes	****	< 0.0001
5 vs. 10 mM	−0.22	−0.3109 to −0.1291	Yes	****	< 0.0001
5 vs. 25 mM	−0.41	−0.5009 to −0.3191	Yes	****	< 0.0001
5 vs. 50 mM	−0.45	−0.5409 to −0.3591	Yes	****	< 0.0001
10 vs. 25 mM	−0.19	−0.2809 to −0.09909	Yes	****	< 0.0001
10 vs. 50 mM	−0.23	−0.3209 to −0.1391	Yes	****	< 0.0001
25 vs. 50 mM	−0.04	−0.1309 to 0.05091	No	ns	0.7414

2. Second (enzymatic) crosslinking step

2.5 mM GTA Gels

- Instantaneous modulus (E_{inst})

	**Day 0**		**Day 1**		**Day 4**		**Day 7**	
	**Mean (Pa)**	**SEM (Pa)**	**Mean (Pa)**	**SEM (Pa)**	**Mean (Pa)**	**SEM (Pa)**	**Mean (Pa)**	**SEM (Pa)**
**0 U/g**	4604	60	3439	39	2170	17	3909	129
**1 U/g**	-	-	31810	115	35850	139	26835	151
**10 U/g**	-	-	59874	322	62413	503	59455	573
**100 U/g**	-	-	62734	356	84134	228	82258	436

Tukey's multiple comparison test					
	**Mean Diff**	**95% CI of diff**	**Significant?**	**Summary**	**Adjusted P Value**
**Day 0**					
**0 vs. 1 U/g**	0	−975.3 to 975.3	No	ns	> 0.9999
**0 vs. 10 U/g**	0	−975.3 to 975.3	No	ns	> 0.9999
**0 vs. 100 U/g**	0	−975.3 to 975.3	No	ns	> 0.9999
**1 vs. 10 U/g**	0	−975.3 to 975.3	No	ns	> 0.9999
**1 vs. 100 U/g**	0	−975.3 to 975.3	No	ns	> 0.9999
**10 vs. 100 U/g**	0	−975.3 to 975.3	No	ns	> 0.9999
**Day 1**					
**0 vs. 1 U/g**	−28371	−29346 to −27396	Yes	****	< 0.0001

**0 vs. 10 U/g**	−56435	−57411 to −55460	Yes	****	< 0.0001
**0 vs. 100 U/g**	−59295	−60270 to −58320	Yes	****	< 0.0001
**1 vs. 10 U/g**	−28064	−29040 to −27089	Yes	****	< 0.0001
**1 vs. 100 U/g**	−30924	−31899 to −29949	Yes	****	< 0.0001
**10 vs. 100 U/g**	−2860	−3835 to −1884	Yes	****	< 0.0001
**Day 4**					
**0 vs. 1 U/g**	−33680	−34655 to −32705	Yes	****	< 0.0001
**0 vs. 10 U/g**	−60244	−61219 to −59268	Yes	****	< 0.0001
**0 vs. 100 U/g**	−81964	−82939 to −80989	Yes	****	< 0.0001
**1 vs. 10 U/g**	−26564	−27539 to −25588	Yes	****	< 0.0001
**1 vs. 100 U/g**	−48284	−49259 to −47309	Yes	****	< 0.0001
**10 vs. 100 U/g**	−21720	−22696 to −20745	Yes	****	< 0.0001
**Day 7**					
**0 vs. 1 U/g**	−22926	−23901 to −21951	Yes	****	< 0.0001
**0 vs. 10 U/g**	−55546	−56521 to −54571	Yes	****	< 0.0001
**0 vs. 100 U/g**	−78350	−79325 to −77375	Yes	****	< 0.0001
**1 vs. 10 U/g**	−32620	−33595 to −31645	Yes	****	< 0.0001
**1 vs. 100 U/g**	−55424	−56399 to −54448	Yes	****	< 0.0001
**10 vs. 100 U/g**	−22804	−23779 to −21828	Yes	****	< 0.0001

**Tukey's multiple comparison test**					
	**Mean Diff**	**95% CI of diff**	**Significant?**	**Summary**	**Adjusted P Value**
**0 U/g**					
**0 vs. 1 (day)**	1165	189.7 to 2140	Yes	*	0.0118
**0 vs. 4 (day)**	2434	1459 to 3410	Yes	****	< 0.0001
**0 vs. 7 (day)**	695.4	−279.9 to 1671	No	ns	0.2564
**1 vs. 4 (day)**	1269	294.1 to 2245	Yes	**	0.0048
**1 vs. 7 (day)**	−469.6	−1445 to 505.7	No	ns	0.6004
**4 vs. 7 (day)**	−1739	−2714 to −763.7	Yes	****	< 0.0001
**1 U/g**					
**0 vs. 1 (day)**	−27206	−28181 to −26231	Yes	****	< 0.0001
**0 vs. 4 (day)**	−31246	−32221 to −30270	Yes	****	< 0.0001
**0 vs. 7 (day)**	−22231	−23206 to −21255	Yes	****	< 0.0001
**1 vs. 4 (day)**	−4040	−5015 to −3064	Yes	****	< 0.0001
**1 vs. 7 (day)**	4975	4000 to 5951	Yes	****	< 0.0001
**4 vs. 7 (day)**	9015	8040 to 9990	Yes	****	< 0.0001
**10 U/g**					

**0 vs. 1 (day)**	−55270	−56246 to −54295	Yes	****	< 0.0001
**0 vs. 4 (day)**	−57809	−58785 to −56834	Yes	****	< 0.0001
**0 vs. 7 (day)**	−54851	−55826 to −53876	Yes	****	< 0.0001
**1 vs. 4 (day)**	−2539	−3514 to −1564	Yes	****	< 0.0001
**1 vs. 7 (day)**	419.6	−555.7 to 1395	No	ns	0.6837
**4 vs. 7 (day)**	2958	1983 to 3934	Yes	****	< 0.0001
**100 U/g**					
**0 vs. 1 (day)**	−58130	−59105 to −57155	Yes	****	< 0.0001
**0 vs. 4 (day)**	−79530	−80505 to −78554	Yes	****	< 0.0001
**0 vs. 7 (day)**	−77654	−78630 to −76679	Yes	****	< 0.0001
**1 vs. 4 (day)**	−21400	−22375 to −20424	Yes	****	< 0.0001
**1 vs. 7 (day)**	−19525	−20500 to −18549	Yes	****	< 0.0001
**4 vs. 7 (day)**	1875	899.9 to 2850	Yes	****	< 0.0001

- Equilibrium modulus (E_{eq})

	**Day 0**		**Day 1**		**Day 4**		**Day 7**	
	**Mean (Pa)**	**SEM (Pa)**	**Mean (Pa)**	**SEM (Pa)**	**Mean (Pa)**	**SEM (Pa)**	**Mean (Pa)**	**SEM (Pa)**
**0 U/g**	1564	4	1435	11	1237	4	1238	4
**1 U/g**	-	-	18333	46	18036	62	18036	62
**10 U/g**	-	-	40312	120	40299	398	40299	398
**100 U/g**	-	-	50849	205	50096	87	50096	87

**Tukey's multiple comparison test**					
	**Mean Diff**	**95% CI of diff**	**Significant?**	**Summary**	**Adjusted P Value**
**Day 0**					
**0 vs. 1 U/g**	0	−548.8 to 548.8	No	ns	> 0.9999
**0 vs. 10 U/g**	0	−548.8 to 548.8	No	ns	> 0.9999
**0 vs. 100 U/g**	0	−548.8 to 548.8	No	ns	> 0.9999
**1 vs. 10 U/g**	0	−548.8 to 548.8	No	ns	> 0.9999
**1 vs. 100 U/g**	0	−548.8 to 548.8	No	ns	> 0.9999
**10 vs. 100 U/g**	0	−548.8 to 548.8	No	ns	> 0.9999
**Day 1**					
**0 vs. 1 U/g**	−16898	−17447 to −16350	Yes	****	< 0.0001
**0 vs. 10 U/g**	−38877	−39426 to −38328	Yes	****	< 0.0001
**0 vs. 100 U/g**	−49414	−49963 to −48866	Yes	****	< 0.0001
**1 vs. 10 U/g**	−21979	−22528 to −21430	Yes	****	< 0.0001
**1 vs. 100 U/g**	−32516	−33065 to −31967	Yes	****	< 0.0001
**10 vs. 100 U/g**	−10537	−11086 to −9988	Yes	****	< 0.0001

**Day 4**					
**0 vs. 1 U/g**	−16798	−17347 to −16249	Yes	****	< 0.0001
**0 vs. 10 U/g**	−39061	−39610 to −38512	Yes	****	< 0.0001
**0 vs. 100 U/g**	−48858	−49407 to −48310	Yes	****	< 0.0001
**1 vs. 10 U/g**	−22263	−22812 to −21714	Yes	****	< 0.0001
**1 vs. 100 U/g**	−32061	−32609 to −31512	Yes	****	< 0.0001
**10 vs. 100 U/g**	−9798	−10346 to −9249	Yes	****	< 0.0001
**Day 7**					
**0 vs. 1 U/g**	−16819	−17368 to −16270	Yes	****	< 0.0001
**0 vs. 10 U/g**	−41566	−42115 to −41017	Yes	****	< 0.0001
**0 vs. 100 U/g**	−49916	−50465 to −49367	Yes	****	< 0.0001
**1 vs. 10 U/g**	−24747	−25296 to −24198	Yes	****	< 0.0001
**1 vs. 100 U/g**	−33097	−33646 to −32548	Yes	****	< 0.0001
**10 vs. 100 U/g**	−8350	−8899 to −7801	Yes	****	< 0.0001

**Tukey's multiple comparison test**					
	**Mean Diff**	**95% CI of diff**	**Significant?**	**Summary**	**Adjusted P Value**
**0 U/g**					
**0 vs. 1 (day)**	129	−419.8 to 677.8	No	ns	0.9301
**0 vs. 4 (day)**	326.2	−222.6 to 875.0	No	ns	0.4185
**0 vs. 7 (day)**	670.8	122.0 to 1220	Yes	**	0.0094
**1 vs. 4 (day)**	197.2	−351.6 to 746.0	No	ns	0.7904
**1 vs. 7 (day)**	541.8	−7.046 to 1091	No	ns	0.0545
**4 vs. 7 (day)**	344.6	−204.3 to 893.4	No	ns	0.3688
**1 U/g**					
**0 vs. 1 (day)**	−16769	−17318 to −16221	Yes	****	< 0.0001
**0 vs. 4 (day)**	−16472	−17021 to −15923	Yes	****	< 0.0001
**0 vs. 7 (day)**	−16148	−16697 to −15600	Yes	****	< 0.0001
**1 vs. 4 (day)**	297.7	−251.1 to 846.5	No	ns	0.5005
**1 vs. 7 (day)**	621	72.18 to 1170	Yes	*	0.0193
**4 vs. 7 (day)**	323.3	−225.5 to 872.1	No	ns	0.4267
**10 U/g**					
**0 vs. 1 (day)**	−38748	−39297 to −38199	Yes	****	< 0.0001
**0 vs. 4 (day)**	−38735	−39284 to −38186	Yes	****	< 0.0001

**0 vs. 7 (day)**	−40896	−41444 to −40347	Yes	****	< 0.0001
**1 vs. 4 (day)**	13.42	−535.4 to 562.2	No	ns	> 0.9999
**1 vs. 7 (day)**	−2147	−2696 to −1599	Yes	****	< 0.0001
**4 vs. 7 (day)**	−2161	−2710 to −1612	Yes	****	< 0.0001
**100 U/g**					
**0 vs. 1 (day)**	−49285	−49834 to −48737	Yes	****	< 0.0001
**0 vs. 4 (day)**	−48532	−49081 to −47983	Yes	****	< 0.0001
**0 vs. 7 (day)**	−49245	−49794 to −48697	Yes	****	< 0.0001
**1 vs. 4 (day)**	753.2	204.4 to 1302	Yes	**	0.0025
**1 vs. 7 (day)**	40	−508.8 to 588.8	No	ns	0.9976
**4 vs. 7 (day)**	−713.2	−1262 to −164.4	Yes	**	0.0048

- Relaxation time (τ)

	**Day 0**		**Day 1**		**Day 4**		**Day 7**	
	**Mean (s)**	**SEM (s)**	**Mean (s)**	**SEM (s)**	**Mean (s)**	**SEM (s)**	**Mean (s)**	**SEM (s)**
**0 U/g**	0.37	0.01	0.36	0.01	0.31	0.01	0.18	0.01
**1 U/g**	-	-	0.79	0.01	0.57	0.01	0.36	0.01
**10 U/g**	-	-	0.90	0.03	0.73	0.05	0.50	0.04
**100 U/g**	-	-	1.03	0.09	0.91	0.014	0.62	0.02

**Tukey's multiple comparison test**					
	**Mean Diff**	**95% CI of diff**	**Significant?**	**Summary**	**Adjusted P Value**
**Day 0**					
**0 vs. 1 U/g**	0	−0.1161 to 0.1161	No	ns	> 0.9999
**0 vs. 10 U/g**	0	−0.1161 to 0.1161	No	ns	> 0.9999
**0 vs. 100 U/g**	0	−0.1161 to 0.1161	No	ns	> 0.9999
**1 vs. 10 U/g**	0	−0.1161 to 0.1161	No	ns	> 0.9999
**1 vs. 100 U/g**	0	−0.1161 to 0.1161	No	ns	> 0.9999
**10 vs. 100 U/g**	0	−0.1161 to 0.1161	No	ns	> 0.9999
**Day 1**					
**0 vs. 1 U/g**	−0.4306	−0.5467 to −0.3145	Yes	****	< 0.0001
**0 vs. 10 U/g**	−0.5435	−0.6596 to −0.4274	Yes	****	< 0.0001
**0 vs. 100 U/g**	−0.6685	−0.7846 to −0.5524	Yes	****	< 0.0001
**1 vs. 10 U/g**	−0.1129	−0.2290 to 0.003229	No	ns	0.0602
**1 vs. 100 U/g**	−0.2379	−0.3540 to −0.1218	Yes	****	< 0.0001

**10 vs. 100 U/g**	−0.125	−0.2411 to −0.008940	Yes	*	0.0291
**Day 4**					
**0 vs. 1 U/g**	−0.259	−0.3751 to −0.1429	Yes	****	< 0.0001
**0 vs. 10 U/g**	−0.4213	−0.5374 to −0.3053	Yes	****	< 0.0001
**0 vs. 100 U/g**	−0.6063	−0.7224 to −0.4902	Yes	****	< 0.0001
**1 vs. 10 U/g**	−0.1624	−0.2784 to −0.04626	Yes	**	0.002
**1 vs. 100 U/g**	−0.3473	−0.4634 to −0.2313	Yes	****	< 0.0001
**10 vs. 100 U/g**	−0.185	−0.3011 to −0.06890	Yes	***	0.0003
**Day 7**					
**0 vs. 1 U/g**	−0.1826	−0.2987 to −0.06646	Yes	***	0.0003
**0 vs. 10 U/g**	−0.3167	−0.4328 to −0.2006	Yes	****	< 0.0001
**0 vs. 100 U/g**	−0.437	−0.5531 to −0.3209	Yes	****	< 0.0001
**1 vs. 10 U/g**	−0.1341	−0.2502 to −0.01801	Yes	*	0.0161
**1 vs. 100 U/g**	−0.2544	−0.3705 to −0.1383	Yes	****	< 0.0001
**10 vs. 100 U/g**	−0.1203	−0.2364 to −0.004238	Yes	*	0.0389

**Tukey's multiple comparison test**					
	**Mean Diff**	**95% CI of diff**	**Significant?**	**Summary**	**Adjusted P Value**
**0 U/g**					
**0 vs. 1 (day)**	0.009539	−0.1066 to 0.1256	No	ns	0.9966
**0 vs. 4 (day)**	0.06207	−0.05402 to 0.1782	No	ns	0.5132
**0 vs. 7 (day)**	0.1882	0.07207 to 0.3043	Yes	***	0.0002
**1 vs. 4 (day)**	0.05253	−0.06356 to 0.1686	No	ns	0.6479
**1 vs. 7 (day)**	0.1786	0.06253 to 0.2947	Yes	***	0.0005
**4 vs. 7 (day)**	0.1261	0.009999 to 0.2422	Yes	*	0.0272
**1 U/g**					
**0 vs. 1 (day)**	−0.4211	−0.5371 to −0.3050	Yes	****	< 0.0001
**0 vs. 4 (day)**	−0.1969	−0.3130 to −0.08083	Yes	****	< 0.0001
**0 vs. 7 (day)**	0.005607	−0.1105 to 0.1217	No	ns	0.9993
**1 vs. 4 (day)**	0.2241	0.1080 to 0.3402	Yes	****	< 0.0001
**1 vs. 7 (day)**	0.4267	0.3106 to 0.5428	Yes	****	< 0.0001
**4 vs. 7 (day)**	0.2025	0.08644 to 0.3186	Yes	****	< 0.0001
**10 U/g**					
**0 vs. 1 (day)**	−0.5339	−0.6500 to −0.4178	Yes	****	< 0.0001
**0 vs. 4 (day)**	−0.3593	−0.4754 to −0.2432	Yes	****	< 0.0001
**0 vs. 7 (day)**	−0.1285	−0.2446 to −0.01240	Yes	*	0.0233
**1 vs. 4 (day)**	0.1746	0.05855 to 0.2907	Yes	***	0.0007

**1 vs. 7 (day)**	0.4054	0.2893 to 0.5215	Yes	****	< 0.0001
**4 vs. 7 (day)**	0.2308	0.1147 to 0.3469	Yes	****	< 0.0001
**100 U/g**					
**0 vs. 1 (day)**	−0.659	−0.7750 to −0.5429	Yes	****	< 0.0001
**0 vs. 4 (day)**	−0.5443	−0.6604 to −0.4282	Yes	****	< 0.0001
**0 vs. 7 (day)**	−0.2488	−0.3649 to −0.1327	Yes	****	< 0.0001
**1 vs. 4 (day)**	0.1147	−0.001409 to 0.2308	No	ns	0.0542
**1 vs. 7 (day)**	0.4101	0.2940 to 0.5262	Yes	****	< 0.0001
**4 vs. 7 (day)**	0.2954	0.1793 to 0.4115	Yes	****	< 0.0001

5 mM GTA Gels

- Instantaneous modulus E_{inst}

	**Day 0**		**Day 1**		**Day 4**		**Day 7**	
	**Mean (Pa)**	**SEM (Pa)**	**Mean (Pa)**	**SEM (Pa)**	**Mean (Pa)**	**SEM (Pa)**	**Mean (Pa)**	**SEM (Pa)**
**0 U/g**	13920	111	13184	83	12603	72	11344	64
**1 U/g**	-	-	38898	310	35559	322	34935	538
**10 U/g**	-	-	74813	529	75029	1289	85375	1397
**100 U/g**	-	-	86294	468	88376	602	104550	1632

**Tukey's multiple comparison test**					
	**Mean Diff**	**95% CI of diff**	**Significant?**	**Summary**	**Adjusted P Value**
**Day 0**					
**0 vs. 1 U/g**	0	−2530 to 2530	No	ns	> 0.9999
**0 vs. 10 U/g**	0	−2530 to 2530	No	ns	> 0.9999
**0 vs. 100 U/g**	0	−2530 to 2530	No	ns	> 0.9999
**1 vs. 10 U/g**	0	−2530 to 2530	No	ns	> 0.9999
**1 vs. 100 U/g**	0	−2530 to 2530	No	ns	> 0.9999
**10 vs. 100 U/g**	0	−2530 to 2530	No	ns	> 0.9999
**Day 1**					
**0 vs. 1 U/g**	−25714	−28243 to −23184	Yes	****	< 0.0001
**0 vs. 10 U/g**	−61629	−64159 to −59099	Yes	****	< 0.0001
**0 vs. 100 U/g**	−73110	−75640 to −70580	Yes	****	< 0.0001
**1 vs. 10 U/g**	−35915	−38445 to −33386	Yes	****	< 0.0001
**1 vs. 100 U/g**	−47396	−49926 to −44866	Yes	****	< 0.0001
**10 vs. 100 U/g**	−11481	−14011 to −8951	Yes	****	< 0.0001
**Day 4**					
**0 vs. 1 U/g**	−22955	−25485 to −20425	Yes	****	< 0.0001

**0 vs. 10 U/g**	−62426	−64956 to −59896	Yes	****	< 0.0001
**0 vs. 100 U/g**	−75773	−78303 to −73243	Yes	****	< 0.0001
**1 vs. 10 U/g**	−39471	−42001 to −36941	Yes	****	< 0.0001
**1 vs. 100 U/g**	−52818	−55348 to −50288	Yes	****	< 0.0001
**10 vs. 100 U/g**	−13347	−15877 to −10817	Yes	****	< 0.0001
**Day 7**					
**0 vs. 1 U/g**	−23592	−26122 to −21062	Yes	****	< 0.0001
**0 vs. 10 U/g**	−74032	−76561 to −71502	Yes	****	< 0.0001
**0 vs. 100 U/g**	−93206	−95736 to −90676	Yes	****	< 0.0001
**1 vs. 10 U/g**	−50440	−52970 to −47910	Yes	****	< 0.0001
**1 vs. 100 U/g**	−69614	−72144 to −67084	Yes	****	< 0.0001
**10 vs. 100 U/g**	−19174	−21704 to −16644	Yes	****	< 0.0001

**Tukey's multiple comparison test**					
	**Mean Diff**	**95% CI of diff**	**Significant?**	**Summary**	**Adjusted P Value**
**0 U/g**					
**0 vs. 1 (day)**	735.8	−1794 to 3266	No	ns	0.8765
**0 vs. 4 (day)**	1316	−1213 to 3846	No	ns	0.5365
**0 vs. 7 (day)**	2576	46.32 to 5106	Yes	*	0.0441
**1 vs. 4 (day)**	580.6	−1949 to 3111	No	ns	0.9345
**1 vs. 7 (day)**	1840	−689.5 to 4370	No	ns	0.2398
**4 vs. 7 (day)**	1260	−1270 to 3790	No	ns	0.5734
**1 U/g**					
**0 vs. 1 (day)**	−24978	−27508 to −22448	Yes	****	< 0.0001
**0 vs. 4 (day)**	−21639	−24169 to −19109	Yes	****	< 0.0001
**0 vs. 7 (day)**	−21015	−23545 to −18486	Yes	****	< 0.0001
**1 vs. 4 (day)**	3339	809.2 to 5869	Yes	**	0.004
**1 vs. 7 (day)**	3962	1432 to 6492	Yes	***	0.0004
**4 vs. 7 (day)**	623.1	−1907 to 3153	No	ns	0.9206
**10 U/g**					
**0 vs. 1 (day)**	−60893	−63423 to −58363	Yes	****	< 0.0001
**0 vs. 4 (day)**	−61109	−63639 to −58579	Yes	****	< 0.0001
**0 vs. 7 (day)**	−71455	−73985 to −68925	Yes	****	< 0.0001
**1 vs. 4 (day)**	−216.1	−2746 to 2314	No	ns	0.9962
**1 vs. 7 (day)**	−10562	−13092 to −8032	Yes	****	< 0.0001
**4 vs. 7 (day)**	−10346	−12876 to −7816	Yes	****	< 0.0001
**100 U/g**					

**0 vs. 1 (day)**	−72374	−74904 to −69844	Yes	****	< 0.0001
**0 vs. 4 (day)**	−74456	−76986 to −71926	Yes	****	< 0.0001
**0 vs. 7 (day)**	−90630	−93160 to −88100	Yes	****	< 0.0001
**1 vs. 4 (day)**	−2082	−4612 to 447.6	No	ns	0.1474
**1 vs. 7 (day)**	−18256	−20786 to −15726	Yes	****	< 0.0001
**4 vs. 7 (day)**	−16173	−18703 to −13644	Yes	****	< 0.0001

- Equilibrium modulus (E_{eq})

	**Day 0**		**Day 1**		**Day 4**		**Day 7**	
	**Mean (Pa)**	**SEM (Pa)**	**Mean (Pa)**	**SEM (Pa)**	**Mean (Pa)**	**SEM (Pa)**	**Mean (Pa)**	**SEM (Pa)**
**0 U/g**	8480	68	8596	21	7667	39	7487	17
**1 U/g**	-	-	27569	119	24678	226	25104	268
**10 U/g**	-	-	52427	293	53556	943	54651	933
**100 U/g**	-	-	63024	280	67015	226	69493	1176

**Tukey's multiple comparison test**					
	**Mean Diff**	**95% CI of diff**	**Significant?**	**Summary**	**Adjusted P Value**
**Day 0**					
**0 vs. 1 U/g**	0	−1710 to 1710	No	ns	> 0.9999
**0 vs. 10 U/g**	0	−1710 to 1710	No	ns	> 0.9999
**0 vs. 100 U/g**	0	−1710 to 1710	No	ns	> 0.9999
**1 vs. 10 U/g**	0	−1710 to 1710	No	ns	> 0.9999
**1 vs. 100 U/g**	0	−1710 to 1710	No	ns	> 0.9999
**10 vs. 100 U/g**	0	−1710 to 1710	No	ns	> 0.9999
**Day 1**					
**0 vs. 1 U/g**	−18973	−20683 to −17262	Yes	****	< 0.0001
**0 vs. 10 U/g**	−43831	−45542 to −42121	Yes	****	< 0.0001
**0 vs. 100 U/g**	−54428	−56139 to −52718	Yes	****	< 0.0001
**1 vs. 10 U/g**	−24858	−26569 to −23148	Yes	****	< 0.0001
**1 vs. 100 U/g**	−35456	−37166 to −33745	Yes	****	< 0.0001
**10 vs. 100 U/g**	−10597	−12308 to −8887	Yes	****	< 0.0001
**Day 4**					
**0 vs. 1 U/g**	−17011	−18721 to −15301	Yes	****	< 0.0001
**0 vs. 10 U/g**	−45889	−47599 to −44179	Yes	****	< 0.0001
**0 vs. 100 U/g**	−59347	−61058 to −57637	Yes	****	< 0.0001
**1 vs. 10 U/g**	−28878	−30588 to −27168	Yes	****	< 0.0001
**1 vs. 100 U/g**	−42336	−44047 to −40626	Yes	****	< 0.0001
**10 vs. 100 U/g**	−13458	−15169 to −11748	Yes	****	< 0.0001

**Day 7**					
**0 vs. 1 U/g**	−17616	−19327 to −15906	Yes	****	< 0.0001
**0 vs. 10 U/g**	−47163	−48874 to −45453	Yes	****	< 0.0001
**0 vs. 100 U/g**	−62006	−63716 to −60295	Yes	****	< 0.0001
**1 vs. 10 U/g**	−29547	−31258 to −27837	Yes	****	< 0.0001
**1 vs. 100 U/g**	−44389	−46100 to −42679	Yes	****	< 0.0001
**10 vs. 100 U/g**	−14842	−16552 to −13132	Yes	****	< 0.0001

**Tukey's multiple comparison test**					
	**Mean Diff**	**95% CI of diff**	**Significant?**	**Summary**	**Adjusted P Value**
**0 U/g**					
**0 vs. 1 (day)**	−116.1	−1826 to 1594	No	ns	0.9981
**0 vs. 4 (day)**	812.6	−897.7 to 2523	No	ns	0.6109
**0 vs. 7 (day)**	992.5	−717.8 to 2703	No	ns	0.4403
**1 vs. 4 (day)**	928.7	−781.6 to 2639	No	ns	0.4996
**1 vs. 7 (day)**	1109	−601.7 to 2819	No	ns	0.3399
**4 vs. 7 (day)**	179.9	−1530 to 1890	No	ns	0.993
**1 U/g**					
**0 vs. 1 (day)**	−19089	−20799 to −17379	Yes	****	< 0.0001
**0 vs. 4 (day)**	−16198	−17909 to −14488	Yes	****	< 0.0001
**0 vs. 7 (day)**	−16624	−18334 to −14913	Yes	****	< 0.0001
**1 vs. 4 (day)**	2891	1180 to 4601	Yes	****	< 0.0001
**1 vs. 7 (day)**	2465	754.8 to 4175	Yes	**	0.0013
**4 vs. 7 (day)**	−425.4	−2136 to 1285	No	ns	0.9184
**10 U/g**					
**0 vs. 1 (day)**	−43947	−45658 to −42237	Yes	****	< 0.0001
**0 vs. 4 (day)**	−45076	−46787 to −43366	Yes	****	< 0.0001
**0 vs. 7 (day)**	−46171	−47881 to −44461	Yes	****	< 0.0001
**1 vs. 4 (day)**	−1129	−2839 to 581.2	No	ns	0.3235
**1 vs. 7 (day)**	−2224	−3934 to −513.4	Yes	**	0.0048
**4 vs. 7 (day)**	−1095	−2805 to 615.7	No	ns	0.3513
**100 U/g**					
**0 vs. 1 (day)**	−54545	−56255 to −52834	Yes	****	< 0.0001
**0 vs. 4 (day)**	−58535	−60245 to −56824	Yes	****	< 0.0001
**0 vs. 7 (day)**	−61013	−62723 to −59303	Yes	****	< 0.0001
**1 vs. 4 (day)**	−3990	−5700 to −2280	Yes	****	< 0.0001
**1 vs. 7 (day)**	−6469	−8179 to −4758	Yes	****	< 0.0001

**4 vs. 7 (day)**	−2479	−4189 to −768.2	Yes	**	0.0012

- Relaxation time (τ)

	**Day 0**		**Day 1**		**Day 4**		**Day 7**	
	**Mean (s)**	**SEM (s)**	**Mean (s)**	**SEM (s)**	**Mean (s)**	**SEM (s)**	**Mean (s)**	**SEM (s)**
**0 U/g**	0.62	0.03	0.65	0.02	0.52	0.02	0.36	0.01
**1 U/g**	-	-	0.89	0.05	0.71	0.06	0.48	0.06
**10 U/g**	-	-	1.38	0.08	0.97	0.05	0.65	0.08
**100 U/g**	-	-	1.38	0.08	1.07	0.06	0.74	0.10

**Tukey's multiple comparison test**					
	**Mean Diff**	**95% CI of diff**	**Significant?**	**Summary**	**Adjusted P Value**
**Day 0**					
**0 vs. 1 U/g**	0	−0.2099 to 0.2099	No	ns	> 0.9999
**0 vs. 10 U/g**	0	−0.2099 to 0.2099	No	ns	> 0.9999
**0 vs. 100 U/g**	0	−0.2099 to 0.2099	No	ns	> 0.9999
**1 vs. 10 U/g**	0	−0.2099 to 0.2099	No	ns	> 0.9999
**1 vs. 100 U/g**	0	−0.2099 to 0.2099	No	ns	> 0.9999
**10 vs. 100 U/g**	0	−0.2099 to 0.2099	No	ns	> 0.9999
**Day 1**					
**0 vs. 1 U/g**	−0.2441	−0.4540 to −0.03417	Yes	*	0.0152
**0 vs. 10 U/g**	−0.7348	−0.9447 to −0.5249	Yes	****	< 0.0001
**0 vs. 100 U/g**	−0.7326	−0.9425 to −0.5226	Yes	****	< 0.0001
**1 vs. 10 U/g**	−0.4907	−0.7006 to −0.2808	Yes	****	< 0.0001
**1 vs. 100 U/g**	−0.4885	−0.6984 to −0.2785	Yes	****	< 0.0001
**10 vs. 100 U/g**	0.002233	−0.2077 to 0.2122	No	ns	> 0.9999
**Day 4**					
**0 vs. 1 U/g**	−0.1873	−0.3972 to 0.02264	No	ns	0.0994
**0 vs. 10 U/g**	−0.4444	−0.6544 to −0.2345	Yes	****	< 0.0001
**0 vs. 100 U/g**	−0.5473	−0.7572 to −0.3373	Yes	****	< 0.0001
**1 vs. 10 U/g**	−0.2571	−0.4671 to −0.04722	Yes	**	0.0092
**1 vs. 100 U/g**	−0.36	−0.5699 to −0.1501	Yes	****	< 0.0001
**10 vs. 100 U/g**	−0.1028	−0.3128 to 0.1071	No	ns	0.5866
**Day 7**					
**0 vs. 1 U/g**	−0.1174	−0.3273 to 0.09251	No	ns	0.4734
**0 vs. 10 U/g**	−0.2866	−0.4966 to −0.07671	Yes	**	0.0027
**0 vs. 100 U/g**	−0.3802	−0.5901 to −0.1703	Yes	****	< 0.0001

**1 vs. 10 U/g**	−0.1692	−0.3791 to 0.04070	No	ns	0.1616
**1 vs. 100 U/g**	−0.2628	−0.4727 to −0.05287	Yes	**	0.0073
**10 vs. 100 U/g**	−0.09357	−0.3035 to 0.1164	No	ns	0.6588

**Tukey's multiple comparison test**					
	**Mean Diff**	**95% CI of diff**	**Significant?**	**Summary**	**Adjusted P Value**
**0 U/g**					
**0 vs. 1 (day)**	−0.03041	−0.2403 to 0.1795	No	ns	0.9822
**0 vs. 4 (day)**	0.09698	−0.1129 to 0.3069	No	ns	0.6324
**0 vs. 7 (day)**	0.2556	0.04568 to 0.4655	Yes	**	0.0097
**1 vs. 4 (day)**	0.1274	−0.08253 to 0.3373	No	ns	0.3997
**1 vs. 7 (day)**	0.286	0.07609 to 0.4959	Yes	**	0.0027
**4 vs. 7 (day)**	0.1586	−0.05130 to 0.3685	No	ns	0.2093
**1 U/g**					
**0 vs. 1 (day)**	−0.2745	−0.4844 to −0.06458	Yes	**	0.0045
**0 vs. 4 (day)**	−0.0903	−0.3002 to 0.1196	No	ns	0.6839
**0 vs. 7 (day)**	0.1382	−0.07173 to 0.3481	No	ns	0.326
**1 vs. 4 (day)**	0.1842	−0.02572 to 0.3941	No	ns	0.1084
**1 vs. 7 (day)**	0.4127	0.2028 to 0.6226	Yes	****	< 0.0001
**4 vs. 7 (day)**	0.2285	0.01857 to 0.4384	Yes	*	0.0267
**10 U/g**					
**0 vs. 1 (day)**	−0.7652	−0.9751 to −0.5553	Yes	****	< 0.0001
**0 vs. 4 (day)**	−0.3474	−0.5574 to −0.1375	Yes	***	0.0001
**0 vs. 7 (day)**	−0.03103	−0.2410 to 0.1789	No	ns	0.9811
**1 vs. 4 (day)**	0.4178	0.2078 to 0.6277	Yes	****	< 0.0001
**1 vs. 7 (day)**	0.7342	0.5243 to 0.9441	Yes	****	< 0.0001
**4 vs. 7 (day)**	0.3164	0.1065 to 0.5263	Yes	***	0.0007
**100 U/g**					
**0 vs. 1 (day)**	−0.763	−0.9729 to −0.5531	Yes	****	< 0.0001
**0 vs. 4 (day)**	−0.4503	−0.6602 to −0.2404	Yes	****	< 0.0001
**0 vs. 7 (day)**	−0.1246	−0.3345 to 0.08533	No	ns	0.4198
**1 vs. 4 (day)**	0.3127	0.1028 to 0.5226	Yes	***	0.0008
**1 vs. 7 (day)**	0.6384	0.4285 to 0.8483	Yes	****	< 0.0001
**4 vs. 7 (day)**	0.3257	0.1158 to 0.5356	Yes	***	0.0004

10 mM GTA Gels

- Instantaneous modulus E_{inst}

	**Day 0**		**Day 1**		**Day 4**		**Day 7**	
	**Mean (Pa)**	**SEM (Pa)**	**Mean (Pa)**	**SEM (Pa)**	**Mean (Pa)**	**SEM (Pa)**	**Mean (Pa)**	**SEM (Pa)**
**0 U/g**	29090	166	28875	221	27610	115	26789	148
**1 U/g**	-	-	62818	277	60399	2091	74821	498
**10 U/g**	-	-	81576	347	94723	653	115954	1118
**100 U/g**	-	-	67786	468	106552	1256	103745	6838

**Tukey's multiple comparison test**					
	**Mean Diff**	**95% CI of diff**	**Significant?**	**Summary**	**Adjusted P Value**
**Day 0**					
**0 vs. 1 U/g**	0	−6779 to 6779	No	ns	> 0.9999
**0 vs. 10 U/g**	0	−6779 to 6779	No	ns	> 0.9999
**0 vs. 100 U/g**	0	−6779 to 6779	No	ns	> 0.9999
**1 vs. 10 U/g**	0	−6779 to 6779	No	ns	> 0.9999
**1 vs. 100 U/g**	0	−6779 to 6779	No	ns	> 0.9999
**10 vs. 100 U/g**	0	−6779 to 6779	No	ns	> 0.9999
**Day 1**					
**0 vs. 1 U/g**	−33943	−40721 to −27164	Yes	****	< 0.0001
**0 vs. 10 U/g**	−52701	−59479 to −45922	Yes	****	< 0.0001
**0 vs. 100 U/g**	−38911	−45689 to −32132	Yes	****	< 0.0001
**1 vs. 10 U/g**	−18758	−25536 to −11979	Yes	****	< 0.0001
**1 vs. 100 U/g**	−4968	−11747 to 1811	No	ns	0.2337
**10 vs. 100 U/g**	13790	7011 to 20568	Yes	****	< 0.0001
**Day 4**					
**0 vs. 1 U/g**	−32790	−39568 to −26011	Yes	****	< 0.0001
**0 vs. 10 U/g**	−67113	−73892 to −60335	Yes	****	< 0.0001
**0 vs. 100 U/g**	−78942	−85721 to −72163	Yes	****	< 0.0001
**1 vs. 10 U/g**	−34324	−41102 to −27545	Yes	****	< 0.0001
**1 vs. 100 U/g**	−46152	−52931 to −39374	Yes	****	< 0.0001
**10 vs. 100 U/g**	−11829	−18607 to −5050	Yes	****	< 0.0001
**Day 7**					
**0 vs. 1 U/g**	−48032	−54811 to −41254	Yes	****	< 0.0001
**0 vs. 10 U/g**	−89165	−95943 to −82386	Yes	****	< 0.0001
**0 vs. 100 U/g**	−76957	−83735 to −70178	Yes	****	< 0.0001
**1 vs. 10 U/g**	−41133	−47911 to −34354	Yes	****	< 0.0001
**1 vs. 100 U/g**	−28924	−35703 to −22146	Yes	****	< 0.0001
**10 vs. 100 U/g**	12208	5430 to 18987	Yes	****	< 0.0001

Tukey's multiple comparison test					
	**Mean Diff**	**95% CI of diff**	**Significant?**	**Summary**	**Adjusted P Value**
**0 U/g**					
**0 vs. 1 (day)**	214.9	−6564 to 6993	No	ns	0.9998
**0 vs. 4 (day)**	1480	−5299 to 8259	No	ns	0.9429
**0 vs. 7 (day)**	2301	−4477 to 9080	No	ns	0.8175
**1 vs. 4 (day)**	1265	−5513 to 8044	No	ns	0.9632
**1 vs. 7 (day)**	2086	−4692 to 8865	No	ns	0.8572
**4 vs. 7 (day)**	821.1	−5957 to 7600	No	ns	0.9894
**1 U/g**					
**0 vs. 1 (day)**	−33728	−40506 to −26949	Yes	****	< 0.0001
**0 vs. 4 (day)**	−31310	−38088 to −24531	Yes	****	< 0.0001
**0 vs. 7 (day)**	−45731	−52510 to −38953	Yes	****	< 0.0001
**1 vs. 4 (day)**	2418	−4360 to 9197	No	ns	0.7941
**1 vs. 7 (day)**	−12003	−18782 to −5225	Yes	****	< 0.0001
**4 vs. 7 (day)**	−14421	−21200 to −7643	Yes	****	< 0.0001
**10 U/g**					
**0 vs. 1 (day)**	−52486	−59264 to −45707	Yes	****	< 0.0001
**0 vs. 4 (day)**	−65633	−72412 to −58855	Yes	****	< 0.0001
**0 vs. 7 (day)**	−86864	−93642 to −80085	Yes	****	< 0.0001
**1 vs. 4 (day)**	−13148	−19926 to −6369	Yes	****	< 0.0001
**1 vs. 7 (day)**	−34378	−41157 to −27600	Yes	****	< 0.0001
**4 vs. 7 (day)**	−21231	−28009 to −14452	Yes	****	< 0.0001
**100 U/g**					
**0 vs. 1 (day)**	−38696	−45474 to −31917	Yes	****	< 0.0001
**0 vs. 4 (day)**	−77462	−84241 to −70683	Yes	****	< 0.0001
**0 vs. 7 (day)**	−74656	−81434 to −67877	Yes	****	< 0.0001
**1 vs. 4 (day)**	−38766	−45545 to −31988	Yes	****	< 0.0001
**1 vs. 7 (day)**	−35960	−42738 to −29181	Yes	****	< 0.0001
**4 vs. 7 (day)**	2807	−3972 to 9585	No	ns	0.7093

- Equilibrium modulus (E_{eq})

	**Day 0**		**Day 1**		**Day 4**		**Day 7**	
	**Mean (Pa)**	**SEM (Pa)**	**Mean (Pa)**	**SEM (Pa)**	**Mean (Pa)**	**SEM (Pa)**	**Mean (Pa)**	**SEM (Pa)**
**0 U/g**	19165	43	19699	67	17709	30	16817	24
**1 U/g**	-	-	43271	91	43054	1709	44303	211
**10 U/g**	-	-	64867	228	67267	410	68333	570

**100 U/g**	-	-	58810	230	61027	962	64968	5872

**Tukey's multiple comparison test**					
	**Mean Diff**	**95% CI of diff**	**Significant?**	**Summary**	**Adjusted P Value**
**Day 0**					
**0 vs. 1 U/g**	0	−5695 to 5695	No	ns	> 0.9999
**0 vs. 10 U/g**	0	−5695 to 5695	No	ns	> 0.9999
**0 vs. 100 U/g**	0	−5695 to 5695	No	ns	> 0.9999
**1 vs. 10 U/g**	0	−5695 to 5695	No	ns	> 0.9999
**1 vs. 100 U/g**	0	−5695 to 5695	No	ns	> 0.9999
**10 vs. 100 U/g**	0	−5695 to 5695	No	ns	> 0.9999
**Day 1**					
**0 vs. 1 U/g**	−23573	−29268 to −17878	Yes	****	< 0.0001
**0 vs. 10 U/g**	−45169	−50864 to −39474	Yes	****	< 0.0001
**0 vs. 100 U/g**	−39112	−44807 to −33416	Yes	****	< 0.0001
**1 vs. 10 U/g**	−21596	−27291 to −15901	Yes	****	< 0.0001
**1 vs. 100 U/g**	−15539	−21234 to −9844	Yes	****	< 0.0001
**10 vs. 100 U/g**	6057	362.1 to 11752	Yes	*	0.0321
**Day 4**					
**0 vs. 1 U/g**	−25345	−31040 to −19650	Yes	****	< 0.0001
**0 vs. 10 U/g**	−49559	−55254 to −43864	Yes	****	< 0.0001
**0 vs. 100 U/g**	−43319	−49014 to −37624	Yes	****	< 0.0001
**1 vs. 10 U/g**	−24214	−29909 to −18519	Yes	****	< 0.0001
**1 vs. 100 U/g**	−17974	−23669 to −12279	Yes	****	< 0.0001
**10 vs. 100 U/g**	6240	545.2 to 11935	Yes	*	0.0253
**Day 7**					
**0 vs. 1 U/g**	−27486	−33181 to −21791	Yes	****	< 0.0001
**0 vs. 10 U/g**	−51516	−57211 to −45821	Yes	****	< 0.0001
**0 vs. 100 U/g**	−48151	−53846 to −42456	Yes	****	< 0.0001
**1 vs. 10 U/g**	−24030	−29725 to −18335	Yes	****	< 0.0001
**1 vs. 100 U/g**	−20665	−26360 to −14970	Yes	****	< 0.0001
**10 vs. 100 U/g**	3365	−2330 to 9060	No	ns	0.4239

**Tukey's multiple comparison test**					
	**Mean Diff**	**95% CI of diff**	**Significant?**	**Summary**	**Adjusted P Value**
**0 U/g**					
**0 vs. 1 (day)**	−533.8	−6229 to 5161	No	ns	0.995

**0 vs. 4 (day)**	1456	−4239 to 7151	No	ns	0.9122
**0 vs. 7 (day)**	2348	−3347 to 8043	No	ns	0.7119
**1 vs. 4 (day)**	1990	−3705 to 7685	No	ns	0.8042
**1 vs. 7 (day)**	2882	−2813 to 8577	No	ns	0.56
**4 vs. 7 (day)**	892.3	−4803 to 6587	No	ns	0.9777
**1 U/g**					
**0 vs. 1 (day)**	−24107	−29802 to −18412	Yes	****	< 0.0001
**0 vs. 4 (day)**	−23889	−29584 to −18194	Yes	****	< 0.0001
**0 vs. 7 (day)**	−25138	−30833 to −19443	Yes	****	< 0.0001
**1 vs. 4 (day)**	217.7	−5477 to 5913	No	ns	0.9997
**1 vs. 7 (day)**	−1031	−6726 to 4664	No	ns	0.9662
**4 vs. 7 (day)**	−1249	−6944 to 4446	No	ns	0.9422
**10 U/g**					
**0 vs. 1 (day)**	−45702	−51397 to −40007	Yes	****	< 0.0001
**0 vs. 4 (day)**	−48103	−53798 to −42408	Yes	****	< 0.0001
**0 vs. 7 (day)**	−49168	−54863 to −43473	Yes	****	< 0.0001
**1 vs. 4 (day)**	−2400	−8095 to 3295	No	ns	0.6975
**1 vs. 7 (day)**	−3465	−9160 to 2230	No	ns	0.3972
**4 vs. 7 (day)**	−1065	−6760 to 4630	No	ns	0.963
**100 U/g**					
**0 vs. 1 (day)**	−39645	−45340 to −33950	Yes	****	< 0.0001
**0 vs. 4 (day)**	−41863	−47558 to −36168	Yes	****	< 0.0001
**0 vs. 7 (day)**	−45803	−51498 to −40108	Yes	****	< 0.0001
**1 vs. 4 (day)**	−2217	−7912 to 3478	No	ns	0.747
**1 vs. 7 (day)**	−6157	−11852 to −462.3	Yes	*	0.0282
**4 vs. 7 (day)**	−3940	−9635 to 1755	No	ns	0.2822

- Relaxation time (τ)

	**Day 0**		**Day 1**		**Day 4**		**Day 7**	
	**Mean (s)**	**SEM (s)**	**Mean (s)**	**SEM (s)**	**Mean (s)**	**SEM (s)**	**Mean (s)**	**SEM (s)**
**0 U/g**	0.84	0.03	0.76	0.04	0.66	0.01	0.45	0.01
**1 U/g**	-	-	0.93	0.03	0.64	0.07	0.49	0.02
**10 U/g**	-	-	1.28	0.08	0.85	0.06	0.70	0.04
**100 U/g**	-	-	1.33	0.12	0.92	0.07	0.73	0.07

Tukey's multiple comparison test					
	Mean Diff	95% CI of diff	Significant?	Summary	Adjusted P Value
**Day 0**					
**0 vs. 1 U/g**	0	−0.1955 to 0.1955	No	ns	> 0.9999
**0 vs. 10 U/g**	0	−0.1955 to 0.1955	No	ns	> 0.9999
**0 vs. 100 U/g**	0	−0.1955 to 0.1955	No	ns	> 0.9999
**1 vs. 10 U/g**	0	−0.1955 to 0.1955	No	ns	> 0.9999
**1 vs. 100 U/g**	0	−0.1955 to 0.1955	No	ns	> 0.9999
**10 vs. 100 U/g**	0	−0.1955 to 0.1955	No	ns	> 0.9999
**Day 1**					
**0 vs. 1 U/g**	−0.1755	−0.3710 to 0.01995	No	ns	0.096
**0 vs. 10 U/g**	−0.5238	−0.7193 to −0.3283	Yes	****	< 0.0001
**0 vs. 100 U/g**	−0.5713	−0.7668 to −0.3758	Yes	****	< 0.0001
**1 vs. 10 U/g**	−0.3483	−0.5437 to −0.1528	Yes	****	< 0.0001
**1 vs. 100 U/g**	−0.3958	−0.5912 to −0.2003	Yes	****	< 0.0001
**10 vs. 100 U/g**	−0.04751	−0.2430 to 0.1480	No	ns	0.9234
**Day 4**					
**0 vs. 1 U/g**	0.02253	−0.1729 to 0.2180	No	ns	0.9909
**0 vs. 10 U/g**	−0.1907	−0.3861 to 0.004801	No	ns	0.0589
**0 vs. 100 U/g**	−0.2571	−0.4525 to −0.06159	Yes	**	0.0042
**1 vs. 10 U/g**	−0.2132	−0.4087 to −0.01772	Yes	*	0.0263
**1 vs. 100 U/g**	−0.2796	−0.4751 to −0.08412	Yes	**	0.0014
**10 vs. 100 U/g**	−0.06639	−0.2619 to 0.1291	No	ns	0.8173
**Day 7**					
**0 vs. 1 U/g**	−0.04535	−0.2408 to 0.1501	No	ns	0.9325
**0 vs. 10 U/g**	−0.2616	−0.4571 to −0.06611	Yes	**	0.0034
**0 vs. 100 U/g**	−0.2907	−0.4862 to −0.09524	Yes	***	0.0008
**1 vs. 10 U/g**	−0.2162	−0.4117 to −0.02077	Yes	*	0.0234
**1 vs. 100 U/g**	−0.2454	−0.4408 to −0.04989	Yes	**	0.0071
**10 vs. 100 U/g**	−0.02913	−0.2246 to 0.1663	No	ns	0.9807

Tukey's multiple comparison test					
	Mean Diff	95% CI of diff	Significant?	Summary	Adjusted P Value
**0 U/g**					
**0 vs. 1 (day)**	0.07997	−0.1155 to 0.2754	No	ns	0.7169
**0 vs. 4 (day)**	0.1755	−0.01999 to 0.3710	No	ns	0.0961
**0 vs. 7 (day)**	0.3937	0.1982 to 0.5891	Yes	****	< 0.0001

**1 vs. 4 (day)**	0.09551	−0.09996 to 0.2910	No	ns	0.5887
**1 vs. 7 (day)**	0.3137	0.1182 to 0.5092	Yes	***	0.0002
**4 vs. 7 (day)**	0.2182	0.02270 to 0.4136	Yes	*	0.0217
**1 U/g**					
**0 vs. 1 (day)**	−0.09555	−0.2910 to 0.09992	No	ns	0.5884
**0 vs. 4 (day)**	0.198	0.002536 to 0.3935	Yes	*	0.0458
**0 vs. 7 (day)**	0.3483	0.1528 to 0.5438	Yes	****	< 0.0001
**1 vs. 4 (day)**	0.2936	0.09809 to 0.4890	Yes	***	0.0007
**1 vs. 7 (day)**	0.4439	0.2484 to 0.6393	Yes	****	< 0.0001
**4 vs. 7 (day)**	0.1503	−0.04517 to 0.3458	No	ns	0.1959
**10 U/g**					
**0 vs. 1 (day)**	−0.4438	−0.6393 to −0.2483	Yes	****	< 0.0001
**0 vs. 4 (day)**	−0.01519	−0.2107 to 0.1803	No	ns	0.9971
**0 vs. 7 (day)**	0.1321	−0.06340 to 0.3275	No	ns	0.3029
**1 vs. 4 (day)**	0.4286	0.2331 to 0.6241	Yes	****	< 0.0001
**1 vs. 7 (day)**	0.5759	0.3804 to 0.7714	Yes	****	< 0.0001
**4 vs. 7 (day)**	0.1473	−0.04821 to 0.3427	No	ns	0.2117
**100 U/g**					
**0 vs. 1 (day)**	−0.4913	−0.6868 to −0.2958	Yes	****	< 0.0001
**0 vs. 4 (day)**	−0.08158	−0.2771 to 0.1139	No	ns	0.7041
**0 vs. 7 (day)**	0.1029	−0.09253 to 0.2984	No	ns	0.5262
**1 vs. 4 (day)**	0.4097	0.2143 to 0.6052	Yes	****	< 0.0001
**1 vs. 7 (day)**	0.5943	0.3988 to 0.7897	Yes	****	< 0.0001
**4 vs. 7 (day)**	0.1845	−0.01095 to 0.3800	No	ns	0.0722
